# The Nile Rat (*Arvicanthis niloticus*) as a Superior Carbohydrate-Sensitive Model for Type 2 Diabetes Mellitus (T2DM)

**DOI:** 10.3390/nu10020235

**Published:** 2018-02-18

**Authors:** Avinaash Subramaniam, Michelle Landstrom, Alice Luu, K. C. Hayes

**Affiliations:** Department of Biology, Brandeis University, Waltham, MA 02454, USA; avinaash1381@gmail.com (A.S.); mlandstrom@brandeis.edu (M.L.); aluu@brandeis.edu (A.L.)

**Keywords:** metabolic syndrome, type 2 diabetes, animal models, glycemic load, nutrition, Nile rat

## Abstract

Type II diabetes mellitus (T2DM) is a multifactorial disease involving complex genetic and environmental interactions. No single animal model has so far mirrored all the characteristics or complications of diabetes in humans. Since this disease represents a chronic nutritional insult based on a diet bearing a high glycemic load, the ideal model should recapitulate the underlying dietary issues. Most rodent models have three shortcomings: (1) they are genetically or chemically modified to produce diabetes; (2) unlike humans, most require high-fat feeding; (3) and they take too long to develop diabetes. By contrast, Nile rats develop diabetes rapidly (8–10 weeks) with high-carbohydrate (hiCHO) diets, similar to humans, and are protected by high fat (with low glycemic load) intake. This review describes diabetes progression in the Nile rat, including various aspects of breeding, feeding, and handling for best experimental outcomes. The diabetes is characterized by a striking *genetic permissiveness* influencing hyperphagia and hyperinsulinemia; random blood glucose is the best index of disease progression; and kidney failure with chronic morbidity and death are outcomes, all of which mimic uncontrolled T2DM in humans. Non-alcoholic fatty liver disease (NAFLD), also described in diabetic humans, results from hepatic triglyceride and cholesterol accumulation associated with rising blood glucose. Protection is afforded by low glycemic load diets rich in certain fibers or polyphenols. Accordingly, the Nile rat provides a unique opportunity to identify the nutritional factors and underlying genetic and molecular mechanisms that characterize human T2DM.

## 1. Introduction

The current increase in metabolic syndrome (MetS) and type 2 diabetes (T2DM) in the world population emphasizes the urgent need to understand the causes and mechanisms underlying the onset and progression of these metabolic disorders. MetS is characterized by insulin resistance with hyperinsulinemia, hypertension, depressed high-density lipoprotein (HDL), increased visceral adiposity, and fatty liver with increased plasma triglycerides (TG), which lead to rising blood glucose (hyperglycemia) and T2DM if sustained [1,2]. It is generally accepted that carbohydrate (CHO), as the dietary glycemic load, is the prime dietary contributor to the increase in blood glucose and eventual diabetes [3,4,5].

Furthermore, dietary carbohydrate restriction, not fat restriction, produces the greatest reduction in blood glucose in humans, both postprandially as well as other measures of glucose overload such as HbA1c [3,6,7,8,9,10,11,12,13,14]. In 2014, an estimated 422 million people worldwide were living with T2DM, many without knowing of their affliction; this figure quadrupled in 35 years. The greatest prevalence is largely observed in low-income and middle-income countries [15,16,17,18]. The global epidemic of T2DM is especially remarkable in indigenous peoples who ate sustenance diets rich in complex carbohydrates and subsequently adopted a Western lifestyle with diets rich in simple carbohydrates, less fruits and vegetables, and more animal products [19]. The World Health Organization (WHO) reported in 2012 that a total of 3.7 million deaths were attributed to T2DM, making it an epidemic. It is thus vital that we search for effective methods for prevention and treatment. The number of people with prediabetes (defined as fasting blood glucose levels between normoglycemia and diabetes, 100–125 mg/dL) is rapidly increasing worldwide, and it has been estimated that by 2040, 482 million people will be prediabetic [20,21].

T2DM is largely a nutritional problem that requires a nutritional paradigm in a spontaneous model that develops diabetes under normal eating conditions, if all aspects of the problem are to be addressed effectively. Because many questions in T2DM research can only be addressed with invasive procedures that cannot be performed in humans for ethical reasons, it is imperative that appropriate animal models be identified. It is highly desirable to have a reliable nutritional model that effectively mimics all or most aspects of the human disease to better understand its pathogenesis and test potential therapeutic interventions. Few models, however, approach this ideal by adequately mimicking the array of symptoms involved, and even fewer replicate the dietary perturbation associated with the human disease [22,23].

Whereas various models have been developed that exhibit some of the symptoms of MetS and T2DM, few represent true diet-induced diabetes that mimic the disease process in humans [24,25,26,27]. Many models depend on specific genetic or chemical manipulations to induce symptoms, whereas gene x diet interactions (i.e., between susceptibility genes and a diabetogenic nutritional environment) are implicated in the human disease via an interaction similar to the etiology of T2DM and MetS in the Nile rat (*Arvicanthis niloticus*) [28]. This overview compares a selection of commonly used rodent models of MetS and T2DM, including the metagenetic physiology of diabetes in the Nile rat and how this model best fulfills the various criteria necessary for effective study of the onset and progression of the disease.

## 2. Common Rodent Models of MetS and T2DM

Table 1 compares the various risk indicators for MetS in humans against the most widely used rodent models. In brief, only the wild-type Nile rat develops all symptoms of human T2DM and MetS when fed a hiCHO diet bearing a high glycemic load.

### 2.1. Mouse Models

#### 2.1.1. C57BL Mouse Background

The C57BL/6 mouse is the most widely researched inbred mouse strain for diabetes, and has been extensively studied as a genetic background for introducing various mutations/traits. Several substrains exhibiting different phenotypes have been produced from the C57BL/6 founder line, including C57BL/6J (maintained at the Jackson Laboratory, Bar Harbor, ME, USA) and C57BL/6N (at the National Institutes of Health, Bethesda, MD, USA) [49,50].

The C57BL/6J mouse and its variant C57 substrains are widely utilized in numerous genetic and nutritional studies to assess the interplay between genetic background and environmental challenges in the development of T2DM-related disease [49,51,52]. The diabetes-prone C57BLKS/J derived from C57BL/6J has also been an attractive model for studies on diabetes susceptibility as it is a genetic composite between resistant and susceptible strains [53].

Obesity is an important risk factor linked to diabetes in humans. Mice homozygous for obese (Lep^ob^) and diabetes (Lepr^db^) mutations (previously known as ob/ob and db/db mice) are widely studied as models for T2DM [54,55]. These mice have an increased feed efficiency due to their leptin dysfunction, which prevents activation of satiety brain signals. Mutations in the leptin gene (Lep) or its receptor (Lepr) induce hyperphagia, resulting in obesity and diabetes. The genetic background of the strain strongly influences the severity of diabetes in homozygous Lep^ob^ and Lepr^db^ mice. Both these mouse strains manifest profound obesity, chronic hyperglycemia, and pancreatic beta cell atrophy, resulting in hypoinsulinemia in the C57BLKS/J genetic background. However, in the C57BL/6J genetic background, the same homozygous mutations result in only transient hyperglycemia, and the beta cells undergo hypertrophy (not atrophy), thus resulting in hyperinsulinemia, not true T2DM. Such models, however, are atypical of the human experience, in part because T2DM in humans is not a simple disease caused by a single gene defect. Rather, T2DM represents a polygenic (more than 60 genes have been implicated) disorder with the environment (diet) playing an important part in its onset and development. Also, beta cells in humans do not typically become hyperplastic with unlimited insulin production. Accordingly, genetic mutations like those of Lep^ob^ and Lep^db^ make C57BL/6 relatively unsuitable for studying the complex nutritional physiology that characterizes human T2DM, induced by diets with a high glycemic load. Its complexity requires a more nuanced system that mimics human dietary stressors to provide a comprehensive approach. Moreover, leptin deficiency is rare in humans and associated with a host of phenotypes beyond T2DM [56].

##### High-Fat Induced Diabetes in C57BL Mouse

C57BL/6 mice are among the most sensitive to diet (high-fat)-induced obesity (DIO). The C57BL/6-DIO mice originally introduced by Surwit et al. (1988) develop obesity, glucose intolerance, moderate insulin resistance, hyperinsulinemia, and increased blood glucose, with fasting glucose levels 200 mg/dL [55]. Such C57 variants are particularly useful for evaluating phenotypic and genotypic expression of specific genes impacting the disease when induced by high-fat consumption [57]. However, the C57BL/6-DIO mouse is limited in its nutritional aspects, because this model requires a high-fat diet (60% energy as fat) for T2DM induction. Most importantly, it fails to do so when fed a hiCHO diet [52,58,59,60,61]. Although several investigators purport that the C57BL/6 is a good model to study human MetS because it develops the symptoms of insulin resistance, obesity, hyperinsulinemia, hyperglycemia and hypertension when fed a high-fat diet, it remains lean and physically normal when fed low-fat chow or hiCHO diets [54,55,59,62]. By contrast, as noted above, most human studies indicate T2DM is most apt to develop when carbohydrate-rich diets with a high glycemic load are consumed. Replacing dietary carbohydrates with fat or certain fibers reduces both the glycemic load and diabetes incidence in humans and Nile rats [30,85,86].

##### Microbiome and Diet in the C57BL Mouse

Finally, dietary-fat-induced diabetes in C57 mice may have limited application to the gut anatomy and microbiome contributions to whole-body metabolism and beta cell function in humans. Mounting evidence indicates that the gut microbiome plays an important role in the regulation of energy homeostasis through its influence on the efficiency of energy extraction from the diet, and the fat load per se alters the microbiome in mice [63,87]. Furthermore, T2DM is associated with abnormal energy metabolism and low-level chronic inflammation associated with molecules such as lipopolysaccharides and endotoxins released by altered microbiota that result in gut barrier dysfunction, which is affected by diet [88,64].

An altered microbiota (dysbiosis) is typically characterized by an increase in the Firmicutes/Bacteroidetes phylum ratio and decrease in functional bacteria such as Bifidobacteria in T2DM patients [89]. The first evidence of an alteration in the gut microbiome in response to an obese phenotype was demonstrated in genetically obese homozygous C57BL/6J lep^ob^ mice where the mice also revealed an increased Firmicutes/Bacteroidetes ratio [65]. However, because the gut microbiota is greatly influenced by diet composition, diet-induced diabetes in C57 mice may have limited utility for T2DM in humans. For example, the microbiome induced by carbohydrate-rich diets in humans likely differs from the microbiome dictated by the high-fat diet of the mouse. It is also unclear how genetic mutations like those of Lep^ob^ might affect the microbiome.

Furthermore, microbiome studies in this mouse model typically use fasting blood glucose (FBG) and an intra-peritoneal glucose tolerance test (ipGTT) to assess the degree of abnormal glycemia [52]. Applying an ipGTT is not ideal in nutritional studies because it tends to emphasize insulin resistance, and the administered glucose load bypasses the contribution of gut mechanisms controlling carbohydrate digestion and glucose absorption affecting diet-induced diabetes. Also, an interaction of glucose with the gut microbiome would only be evaluated by a carbohydrate gavage interacting with the entire digestive tract. Consequently, the C57BL/6 mouse is a questionable model for evaluating the gut microbiome relative to typical human dietary conditions. Due to the complications and contradictions that arise from these various manipulations, it is preferable to control these complex associations by assessing T2DM in a model where diabetes induction is more typical of the human disease.

#### 2.1.2. Spiny Mouse

The spiny mouse (*Acomys cahirinus*) is a desert rodent that has adapted to conditions of limited caloric supply. However, the laboratory environment with abundant food results in hyperglycemia in this species, making it a potentially interesting spontaneous model for diet-induced diabetes. However, the pathway leading to diabetes is atypical of human T2DM because it is not characterized by insulin resistance, and, again, diabetes is induced with high-fat, not with a hiCHO diet. Fed a carbohydrate (sucrose)-rich diet, the spiny mouse develops marked hepatic lipogenesis and hyperlipidemia and elevation of very low-density lipoproteins (VLDL) without obesity or diabetes, although beta-cell hypertrophy is evident. On a fat-rich diet, however, distinct weight gain and increase in adipose tissue is evident. The obesity is accompanied by hyperinsulinemia and hyperglycemia with glucose intolerance [39,90].

### 2.2. Common Rat Models

As in mice, high-fat feeding is often applied to conventional inbred rats, such as the Sprague Dawley strain, to induce obesity and trigger certain aspects of T2DM. In addition, several more specialized models have been described.

#### 2.2.1. Spontaneously Diabetic Torii (SDT) Rat

The diabetic Torii rat represents an atypical rat model of T2DM specifically related to insulin deficiency, especially for non-obese T2DM. It is an inbred model that spontaneously develops hyperglycemia, glycosuria and glucose intolerance resulting from genetic defects that cause gradual beta-cell degeneration, impaired insulin secretion, and eventual development of the diabetes [73]. SDT rats also spontaneously exhibit the severe ocular complications of retinal degeneration and detachment associated with the chronic hyperglycemia found in humans with diabetes, rendering this model useful for studying diabetic retinopathy [73,74,75,76].

Diabetes in the SDT rat has a polygenic basis, regulated by at least seven quantitative trait loci (QTL) for glucose intolerance: Gisdt1, Gisdt2, and Gisdt3 on rat chromosomes 1, 2 and X and Dmsdt1, Dmsdt2, Dmsdt3 and Dmsdt4 on chromosomes 3, 8, 13 and 14 [73,74,77]. Dmsdt1, was reported to be the major locus responsible for pancreatic lesions in SDT rats and the most influential in the development of diabetes [78]. The numerous studies using the SDT rat have assessed glycemia by oral glucose tolerance test (OGTT), fasting blood glucose (FBG) and random blood glucose (RBG) [74,76].

When fed a commercial pelleted chow (CLEA Rodent Diet CE-2; 63:6:31 CHO:Fat:Protein %energy), male SDT rats exhibited glycosuria and glucose intolerance without obesity at 20 weeks of age. Hyperglycemia, hypoinsulinemia and fibrosis of pancreatic islets occurred after 25 weeks of age, and non-fasting random glucose (RBG) in male SDT rats also reached 700 mg/dL. Hypertriglyceridemia followed at 35 weeks. By 38 weeks 100% of SDT rats spontaneously developed diabetes, and all had cataracts by 40 weeks of age [79].

On the other hand, SDT rats become diabetic more rapidly when fed a hiCHO semi-purified diet (61:14:25; CHO:Fat:Protein %energy, 3.59 kcal/g), again linked to decreased insulin production with age. When fed a hiCHO diet, SDT rats begin developing diabetes at 12 weeks, followed by a rapid rise in blood glucose from 12 to 24 weeks. By contrast, a high-fat diet (18:62:20, 5.04 kcal/g) induced obesity, hyperinsulinemia, and hyperlipidemia between 10 and 16 weeks, but hyperglycemia did not appear until after 16 weeks of age, indicating the hiCHO diet was slightly more diabetogenic.

After 16 weeks of high-fat consumption, the age-dependent progress of hyperglycemia associated with hypoinsulinemia (from insulin deficiency described above) becomes delayed because hyperfunction of pancreatic beta cells induced by the high-fat diet appears to suppress development of the hypoinsulinemia/hyperglycemia. In effect, a high-fat diet improved glucose tolerance and insulin secretion in SDT rats compared to SDT rats fed a standard hiCHO diet. However, serum triglycerides and cholesterol were markedly increased by fat feeding, adding to the complexity of the model. Because insulin resistance and dyslipidemia coexisted with fat-induced obesity, the metabolic response to high-fat intake was concluded to be similar to that observed in other rat or mouse strains, which, again, is inconsistent with the human condition. The FBG, obtained after 4 h of food restriction and prior to glucose loading, as well as the 30 and 60 min-OGTT values, were significantly lower in SDT rats fed high-fat (18:62:20 CHO:Fat:Protein %energy, 5.04 kcal/g) compared to rats fed the standard hiCHO diet (61:14:25, 3.59 kcal/g). Accordingly, the glucose area under the curve (AUC) was also decreased by high-fat intake [76].

Furthermore, a congenic strain of the SDT rat, the SDT-fatty rat (SDT Lepr^fa^), was established by introducing a fatty (fa) allele of the Zucker Diabetic Fatty (ZDF fa/fa ) rat, into the genome of the SDT rat deficient in the leptin receptor gene (Lepr^fa^), such that the homozygous fa/fa strain develops diabetes earlier, from five weeks of age, while heterozygous and wildtype strains develop diabetes at 20 weeks [80]. However, multiple studies have confused this congenic strain with the original SDT rat, making it difficult for the reader to comprehend model differences. Because SDT-fatty rats become obese when fed a high-fat diet as the stressor, and high-fat diet is not considered the main cause of obesity and T2DM in humans, the SDT model lacks integrity related both to mode of diet induction and its severe beta cell failure resulting from dietary fat stress, similar to other diet fat-stressed models [3,9,76].

#### 2.2.2. Zucker Diabetic Fatty (ZDF) Rat

In the Zucker Diabetic Fatty rat (ZDF), a high-fat diet exaggerates hyperglycemia/hypoinsulinemia and ultimately accelerates the exhaustion of beta cells. While rats did develop diabetes on a low-fat diet, the process was accelerated as dietary fat increased, indicating fat aggravated T2DM in the ZDF rat, unlike in the SDT rat, where fat delayed T2DM onset [81]. Thus, a high-fat diet in ZDF rats leads to an elevated risk of lipotoxicity in pancreatic beta cells. The difference in the glycemic response to a high-fat diet between SDT and ZDF rats might be explained by a vulnerability of the pancreatic beta cells to lipid accumulation initiated by high-fat intake in the latter, whereas the SDT rat is more vulnerable to glucose loading that increases insulin demand and leads to beta cell failure and hypoinsulinemia, all of which emphasizes the role of diet composition in diabetes outcomes in these models.

#### 2.2.3. UCDavis-Type 2 Diabetes Mellitus (UCD-T2DM) Rat/ Zucker Diabetic Sprague Dawley (ZDSD or ZDSD/Pco) Rat

The UCD-T2DM and ZDSD rats essentially represent polygenic late-onset obesity with insulin resistance and eventual beta cell insufficiency, while possessing normal leptin signaling. The diabetes evolves after several months of feeding. These two recent rat models both were developed in separate labs in the early 2000s by crossing obese insulin-resistant Sprague Dawley (OSD) rats, to provide fat sensitive genes for obesity and insulin resistance, with the Zucker Diabetic Fatty-lean (ZDF fa +/−) rat, to include a genetic beta cell defect to assure eventual evolution of insulin insufficiency and severe T2DM with certain characteristics of human T2DM [25,26,82,83,84,91].

While these two rat models incorporate key aspects of human T2DM, they are not spontaneous or naturally occurring and thus limit the full range in *genetic permissiveness* associated with regulation of blood glucose. Both rats are positioned as models for drug discovery paradigms to demonstrate diabetes deterrence or reversal using targeted pharma agents, one rat developed in academia (UCD-T2DM), the other in a commercial setting (ZDSD). Although generated from different founder lines with progeny that presumably have diverged over time, they have similar T2DM characteristics, both requiring added fat (to 27% or 48% energy) that preferentially favors diabetes in males. To date, experiments have been conducted with minimal evaluation of the macronutrient interaction with the diabetogenic gene mutations borrowed from prior models. In other words, the objective has generally been to intervene with drug therapy rather than address the underlying dietary components needed for better understanding of diabetes prevention, which is a current world problem.

In fact, both rats are manipulated with fat supplements to induce the diabetes [25,84,91] and in one instance with a fructose supplement at 20% energy mixed with lab chow 5012 (60:13:27 CHO:Fat:Protein %energy) along with safflower oil for a final energy profile of 56:27:17 [92] but without due consideration of the various nutrients being impacted simultaneously. One ZDSD study altered adiposity and diabetes outcome by introducing the large fat supplement (at 48% energy) early in young adult male growing rats, but the high-fat diet also contained marginal protein energy (43:48:9, 6% fiber) than the 5008 chow control (56:17:27, 6% fiber of different type) [84]. Thus, the diets employed to induce diabetes are rather broadly defined nutritionally, for the most part based on lab chow (Lab Diet 5008 or 5012), not semi-purified diets, in which the macronutrient compositions can be controlled and well characterized. Moreover, to date the basic issue of diet x gene interaction that occurs in human T2DM within the context of global dietary changes [21] has not been addressed. This contrasts with the Nile rat studies (see below) where diet composition is the primary focus for its impact on a naïve wildtype genetic background affecting glucose metabolism and designed to clarify which dietary macronutrient(s) are responsible for the diet x gene interactions.

Furthermore, although obesity and T2DM can develop in both sexes of these newer rat models, female ZDSD rats only do so with a high-fat, low-protein diet (43:48:9) [91], while males of both models respond earlier and more robustly to low-fiber lab chows (Lab chow 5012 or 5008) with added fat. However, progression of the disease is slow depending on the levels of added fat [27,82,84,91,92], and the diabetes is not readily attributed to specific macronutrients. For example the high-fat, low-protein diet fed to ZDSD rats altered all macronutrients simultaneously compared to the control 5008 chow (56:17:27) because although the challenge diet reduced CHO modestly by 12% energy, fat was increased by 31% energy, and protein declined by 19% energy to below normal maintenance protein for adult rats. Accordingly, the “diet effect on T2DM” cannot be ascertained, a caveat recently alluded to by others [67]. The point is that one cannot assess whether T2DM markers increased due to the switch to high-fat, the change in glycemic load, or the marked reduction in protein, or a combination of all three. In any case, a high-fat, low-glycemic load diet with extremely low protein energy is not representative of a diet linked to T2DM in humans. As such, it introduces considerable nutritional bias without justification and leaves the reader without a clear understanding of the nutritional attributes impacting diabetes.

#### 2.2.4. Goto-Kakizaki Rat

The Goto-Kakizaki (GK) rat is a polygenic non-obese model for T2DM that develops diabetes on carbohydrate-rich chow diets [68,69]. It is one of the best characterized rodent models of spontaneous T2DM and has several features in common with the disease in humans that make it a valuable tool for studying and exploring therapeutic options of T2DM [70]. The model is characterized by progressive loss of beta cells and spontaneously develops hyperinsulinemia, insulin resistance and hyperglycemia with hyperleptinemia, hyperphagia, increased gluconeogenesis, and accumulation of visceral fat [70,71].

The β-cell failure is linked to prolonged hyperglycemia/hyperlipidemia, associated with inflammation and oxidative stress. Pathophysiological conditions such as insulin resistance, and impaired insulin secretion related to pancreatic islet lesions, are not completely understood in human T2DM owing to ethical and technical challenges in accessing human pancreatic islets. The GK rat may provide an opportunity to investigate this process in more detail. However, one drawback is the confounding effect of high corticosterone, which increases hepatic gluconeogenesis. In fact, their fasting hyperglycemia depends on elevated corticosterone [72]. Although increased hepatic gluconeogenesis is a major contributing factor to fasting hyperglycemia in T2DM in humans, it is not associated with increased levels of corticosterone []. Thus, the underlying cause of hyperglycemia in the GK rat (and several other rodents) does not closely reflect that in humans and may not be relevant in studying mechanisms of diet-induced T2DM or exploring the response to therapeutic agents.

#### 2.2.5. Koletsky Rat

The Spontaneous Hypertensive Obese Rat (SHROB) rat, also known as the Koletsky rat, was developed in 1972 by crossing a Spontaneous Hypertensive rat with a ZDF rat and then a Sprague Dawley rat. As an obese model of T2DM, it carries a nonsense mutation (fa^k^) in the leptin receptor. It displays several phenotypes that reflect MetS, such as obesity, hyperinsulinemia, insulin resistance, hyperlipidemia and hypertension [22,24,31,32]. However, fasting blood glucose is normal, so, despite the several features of MetS, it is not entirely suitable for studying diet-induced human T2DM. Furthermore, as explained earlier, Leptin deficiency is uncommon in humans and associated with a multitude of phenotypes beyond T2DM [56].

### 2.3. Spontaneous T2DM in Rats

#### 2.3.1. Nile Rat and Sand Rat

The process, onset, and progression of T2DM in humans typically represent nutritionally-induced insulin resistance as a consequence of a diet bearing a high glycemic load. This leads eventually to hyperglycemia and insulin deficiency as beta cells fail. The best model of this scenario should adhere to the human paradigm in its etiology and pathophysiology. While the SDT is useful for studying diabetic retinopathy, it is not a true nutritional model of this condition, nor is it faithful to the etiology of human T2DM. A natural spontaneous model with appropriate nutritional and physiological attributes is preferred, if all aspects of disease progression are to be represented accurately. Also, human T2DM is polygenic in origin, but as indicated above many rodent models reflect specific genetic manipulations that induce partial symptoms and fail to account fully for gene x diet interactions that are implicated in the human disease. Only a few models match such criteria by adequately mimicking the array of symptoms involved, and few replicate the dietary perturbations associated with the human disease [22].

The Nile rat, also known as the African grass rat (*Arvicanthis niloticus*), and the Sand rat (*Psammomys obesus*), are North African rodents that appear to be the only two naturally, well documented, presumably polygenic models of diabetes displaying nutritional attributes that mimic the spontaneous pathophysiology of MetS and T2DM in humans, i.e., both models display dietary carbohydrate-induced diabetes without additional manipulation. Also, part of the appeal of these two models is that the underlying genetic profile that allows for their CHO-induced metabolic disturbance and diabetes is still unknown and may shed light on the human condition once identified.

#### 2.3.2. Sand Rat

The Sand rat and Nile rat seem to respond similarly when held in captivity and fed chow [28,33,34,35,38]. Both progress through stages of insulin resistance with hyperinsulinemia, leading to beta cell failure, and finally to severe hyperglycemia characterized by inadequate insulin production and insulin deficiency associated with ketoacidosis resulting in adipose and muscle-wasting disease [33,34,35,38,40,41,42].

As a nutritional model, however, the diabetes has not been adequately resolved in the Sand rat because selective modification of ingredients in semi-purified diets designed to modify individual ingredients has not been reported. Specifically, the carbohydrate, fat, protein nutrient composition has not been detailed sufficiently as a function of the disease process. In addition, blood glucose seems not to have been assessed with RBG, relying primarily on FBG, which has been observed to be too late an index for monitoring the initial stages of diabetes in 10-week Nile rat studies [30]. As detailed above, ipGTT used in most Sand rat studies, introduces a bias because the glucose delivery bypasses the entire gut digestion and absorption mechanisms, an important aspect of naturally occurring diet-induced diabetes. As such the test represents a method to assess glucose clearance and insulin resistance. Weighing organs at necropsy is also a simple method to establish diabetes progression resulting from dietary perturbations, a measure not fully assessed in most descriptions of Sand rat diabetes [39,43,44,45,46,47].

Metabolic disease investigation of animal models, especially for T2DM, ideally utilizes diets with purified or semi-purified ingredients in order to control nutritional variables, because such diets can best minimize variability attributable to diet. However, most studies with the Sand rat have fed two types of plant-based diets to the rat: a pelleted Low Energy, extremely low fat (actually ‘very low energy’ at 68:7:24, 2.32 kcal/g) crude, high fiber rodent diet, and a High Energy diet (40:40:20, 2.92 kcal/g; not high energy as claimed [see below]) extruded rodent maintenance diet.

While the total combustible energy and digestible energy contents from protein, fat, carbohydrates were reported, the detailed composition of the diet constituents and ingredients have not been comprehensively described or delineated in terms of their contribution to T2DM [48]. Furthermore, the so-called “high-energy” diet at 2.92 kcal/g in actuality is a moderate-energy diet, considering that most semi-purified rodent diets range from 3.5 to 4.5 kcal/g or more, suggesting that ‘energy’ itself was not the main consideration for diabetes induction in those Sand rat studies. Fat-enriched, truly high-energy diets (5 kcal/g) have not been examined with emphasis on diabetes to determine how fat might impact the disease in Sand rats.

Consequently, the percentage of calories from each macronutrient has not been well delineated in Sand rat studies, making it difficult to link the energy from different macronutrient classes to the diabetic process. In addition, ingredients in these semi-purified diets in some studies based on plant-based ingredients would have included phytochemicals (e.g., polyphenols, flavonoids) and other minor phytonutrients. These compounds likely contributed undesirable or undisclosed variables, such as phytoestrogens, that can act as endocrine disruptors and affect disease progression. There is also no clear distinction made for diabetes susceptibility between male and female Sand rats, unlike the case for the diabetic permissiveness observed in the male Nile rat [35]. Finally, the fiber content of sand rat diets is typically difficult to ascertain in terms of its soluble-insoluble subclasses, and fiber digestion is known to modulate energy balance in such studies. In fact, it was reported that fiber digestion provided 32% of the Sand rat maintenance energy requirement and 110% of basal metabolic rate (BMR) needs, one of the highest values reported for placental mammals [93]. This likely links its T2DM susceptibility to gut microbiome adaptations impacted by diet fiber content and type. The influence of fiber on the gut flora and the impact of the latter on T2DM is currently a major topic of interest in diabetes research [30,66,94,95,96,97,98]. Not properly controlled, dietary fiber affects the glycemic load and diabetes outcome; and not knowing the fiber-related impact on microbiome function limits understanding and interpretation of the overall dietary impact of the gut flora contribution to diabetes [30,52,99].

Furthermore, unlike humans [36] or Nile rats [100,101], the Sand rat [39] reportedly does not develop diabetic retinopathy during prolonged T2DM.

## 3. The Nile Rat (*Arvicanthis niloticus*)

The following overview emphasizes the utility and preferred attributes of the Nile rat for metabolic studies, especially related to T2DM. Also included are aspects of laboratory housing and husbandry for effective breeding and handling of the model to enhance welfare and minimal handling during experiments. Detailed nutritional studies in the Nile rat have addressed several of the above points and help resolve certain aspects of the nutritional physiology involved, further highlighting the inadequacies of other potential models. The interplay of genetic factors and dietary attributes in the development of T2DM in the Nile rat appears similar to that in humans. For example, the ‘*genetic permissiveness’* of diabetes in human populations is emphasized by the higher susceptibility of certain indigenous populations such as the Pima Indians of the Southwestern United States, Polynesians, and Australian aborigines to T2DM [19,21,102,103].

### 3.1. Nile Rat Background

**Figure 1:**
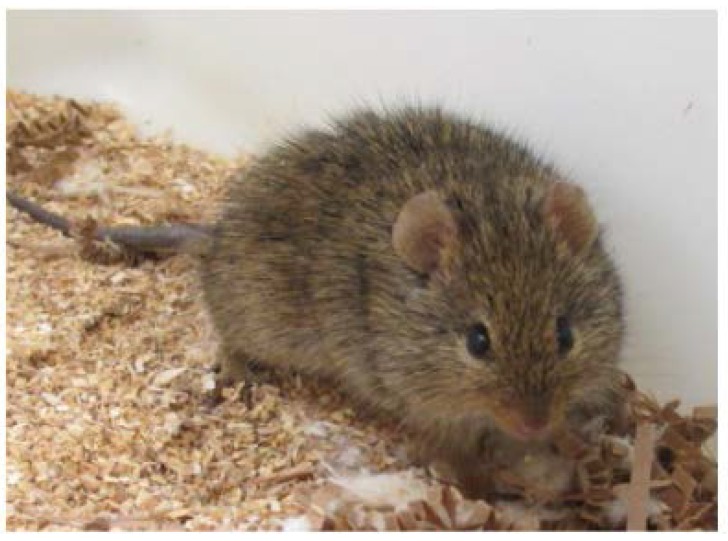
Young adult male Nile rat at approximately 90–100 g bedded on *BetaChip.*

The Nile rat (Figure 1), also known as the African Grass Rat or Nile Grass Rat, is an herbivorous rodent inhabiting the Nile River delta and the savannah grasslands of North Africa. This rodent is highly adaptable to a captive environment, where it breeds well even as it develops metabolic disease. In the wild, where food is a limited resource, the Nile rat primarily consumes fiber-rich native plants in its semi-arid desert environment. Under these circumstances it does not develop diabetes. However, when held in captivity, even the chow-fed rat slowly develops MetS that evolves into diet-induced T2DM with all the pertinent features of the human condition: insulin resistance, hyperinsulinemia, expansion of intra-abdominal fat pools, hypertension, elevated TG with decreased HDL, and eventually hyperglycemia and beta cell failure resulting in depressed insulin and end-stage diabetes that includes severe ketosis [33,35]. The beta cell failure follows the same course as the five-stage decline documented in humans with T2DM, and has been prevented by feeding high-fiber Chinchilla chow [38]. They also represent a useful nutritional model for study of diabetic lesions of the eye, including cataracts and diabetic retinopathy, and as indicated below, advanced diabetes is accompanied by renal failure [34,35,101]. Captive Nile rats have not been reported as harboring any bacteria or viruses known to be harmful to themselves or to other rodents or humans.

Selective breeding colonies of Nile rats exist in North America. The primary colony is maintained at Michigan State University (maintained by Dr. Laura Smale) (East Lansing, MI, USA), who gave rise to the colonies at Brandeis University and the University of Alberta in Canada. Small breeder groups exist at UC Santa Barbara and University of Utah, which originated with breeder stock from the Brandeis colony. Dr. Hayes has several collaborative arrangements in progress globally, and is open to assisting others initiate exploratory projects using catalogued tissues and plasma from the Brandeis nutritional studies and/or to help others start nascent colonies. To our knowledge no commercial vendor has Nile rats for sale at this time. The Nile rats under study at Brandeis University are wild-type and raised under conventional housing conditions for rodents. No rodent or human pathogens, including parasites, bacteria, or viruses, have ever been identified in the colony and, hence, it is seemingly a disease-free rodent colony.

#### 3.1.1. Establishing Breeding Pairs

Nile rats paired for breeding are conventionally housed in 7.5 in × 11.5 in × 5 in animal cages with environmental enrichment devices made from 2 in diameter polyvinyl chloride (PVC) tubes cut into 4 in lengths, nestlets for nesting, and *BetaChip* hardwood bedding in air-conditioned rooms with 12 h light cycle (temperature 68–72 °F, humidity 40–60%). The type of bedding can be a concern because if fiber-free diets are tested for their diabetes impact, rats may consume the bedding as a fiber replacement, which can alter their metabolism. Breeders are fed ad libitum with standard laboratory chow (Lab Diet 5008, 3.3 kcal/g, Purina Mills International, Brentwood, MO, USA) or semi-purified diets placed in a standard stainless-steel cage top with a water bottle always available.

Breeding pairs are usually established at 7–10 weeks after birth in a 1:1 male:female ratio. More than one male in breeding groups is contraindicated because they become territorial and aggressive. Breeding pairs are monitored for birth every weekday morning. Parents with litters typically keep pups in a corner in a pile of shredded nest material (presumably for retaining body heat and security reasons). If potential breeding pairs exhibit violent behavior when paired, they are separated immediately, as delay is often fatal for either gender depending on the aggressor. Pairing rats early, at 6–8 weeks of age, helps acclimate them before sexual maturity and can mitigate aggressiveness during subsequent breeding. Nile rat gestation is about 21–22 days. Nile rat breeding pairs have litters ranging in size from one to eight pups. Younger breeding pairs have larger litters of 4–8 pups on average, and the litter size decreases with age. Pairs are usually productive for about six months, sometimes longer, and probably sustained best in a nondiabetic state with Chinchilla chow, which has a higher fiber content, although to date only Lab Chow 5008 has been fed to breeders at Brandeis [38]. Unpublished data suggest that high-fiber diets during breeding may render the resulting pups less susceptible to T2DM, suggesting that epigenetic factors may be at work during gestation under such conditions.

#### 3.1.2. Separation of Pups

Pups are separated at approximately three weeks of age when weaned at about 25–35 g. If the female gives birth to a new litter before separation of pups has occurred, the older litter is immediately separated if pups weigh 20 g or more. Pregnancy lasts about 21–22 days, and females rebreed soon after delivery, so litters do overlap occasionally. Competition for milk can be extreme, and new pups are often killed and eaten by the mother or older litter. Therefore, it is important to separate pups on schedule. When weaned for study in individual cages, each rat is given a cage card labeled with date of birth, assigned number, parental ID, and gender and fed ad libitum with a semi-purified diet formulated in accord with the study design. If pups are too small to be weaned, they are returned to their parents until they weigh at least 25–30 g. Chow is replaced with semi-purified diets appropriate to the study, and food is replaced every Monday, Wednesday, and Friday, with food intake recorded to monitor caloric consumption during experiments. Water bottles are weighed weekly to record water intake.

##### Growth Curve

Weanling Nile rats at three weeks old weigh 25–35 g. The growth rate can differ depending on the macronutrient composition of the diet fed, with males weighing 75–82 g at seven weeks, 85–95 g at nine weeks, 90–100 g at 11 weeks, and 95–110 g at 13 weeks. Nile rats 1–2 years old can range from 105–150 g, with males larger than females by 15–20 g. Older rats with diabetes undergo weight loss with ketosis, declining to 95–110 g or lower. Retired males seldom live beyond a year, females seldom beyond two years. Retired breeders kept for spontaneous aging are maintained on Lab Chow 5020, which is less diabetogenic (higher fat) than Lab Chow 5008.

#### 3.1.3. Handling the Nile Rat

**Figure 2:**
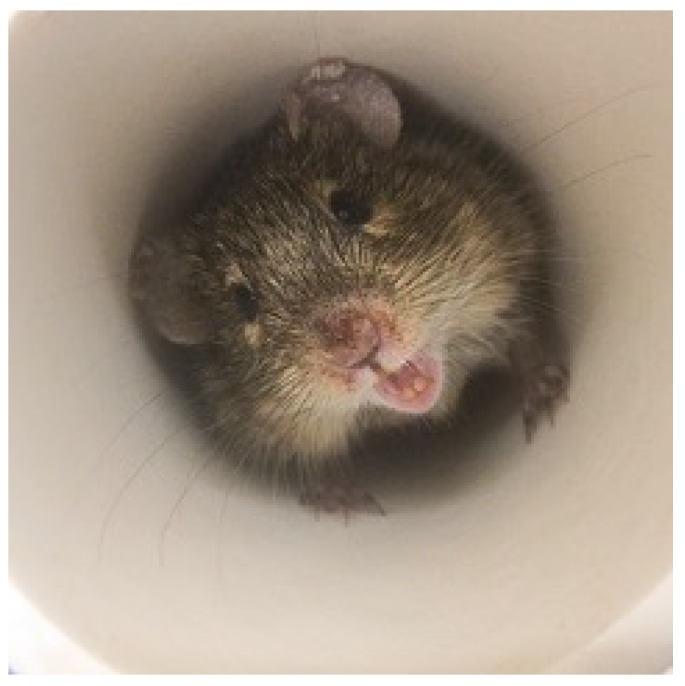
Nile rat in 2 in polyvinyl chloride (PVC) tube for transfer.

Unlike many commercially domesticated lab mice and rats, Nile rats are not tame, which can make researchers hesitant to use them as models. One cannot simply reach into the cage to retrieve them without the risk of being bitten. A specific technique for handling the Nile rats has been developed by applying a 10 in long piece of the 2 in diameter PVC tubing similar to the 6 in one (described earlier) in their cage, but a 2 in diameter rubber stopper closes one end (Figure 2). The open end is placed in front of the Nile rat to encourage it to enter the tube. They become accustomed to this maneuver as pups when cages are changed weekly by animal staff in the breeding colony. Plus, they naturally inhabit subterranean tunnels and burrows in the wild.

If they resist, a small fishnet (also used to recapture escapees) is used to restrict their movement and herd them gently into the tube. Once in the tube, they feel secure and seldom jump out. At this point the rat is lightly anesthetized with 50% CO_2_/50% O_2_ directed into the tube (at 5 mm/Hg flow rate), from a gas tank stationed in the animal or necropsy room. After 15–25 s the rat is sedated, and a 25# needle is used to prick the tail near the mid-region and a commercial Bayer Contour^TM^ glucometer is used to obtain the blood glucose reading. Once blood glucose has been recorded, the gas flow is stopped and the rat returned to its cage to avoid excessive exposure to the gas. The whole procedure, from removing the rat from the cage to its return, takes less than 2 min and is performed without stressing the rat, so as not to distort the glucose value. Nile rats typically regain full consciousness after less than 10 s back in their cage. This same anesthesia method is used prior to necropsy, but the rat is exsanguinated under the anesthesia by cardiac puncture and the chest cavity opened and vena cava severed to prevent recovery.

### 3.2. Diet-Induced T2DM Diabetes in the Nile Rat

#### 3.2.1. Diet Formulation

A commonly applied diet for many conventional rat and mouse studies has been standard laboratory chow, or some variation of it supplemented with fat to render it a ‘high-fat Western diet’. Unlike rats and mice, they are relatively sensitive to dietary cholesterol, so its use must be carefully implemented. One problem with rat chow or its plant-based facsimile, however, is that the macronutrient composition is crudely formulated. Thus, the CHO:Fat:Protein energy ratio and fiber or phytochemical content cannot be precisely documented or interpreted effectively. Hence, the manipulation of individual carbohydrates, fats, or proteins cannot be realized, and interactive effects of nutrients cannot be studied for their impact on diabetes induction. A hiCHO semi-purified diet with a high glycemic load fed to Nile rats is more diabetogenic than rat chow and, hence, diabetes develops more quickly. Insight into the Nile rat glucose response to diet can be further determined by manipulating ratios of macronutrients, fiber, and polyphenol content, as each of these can be controlled and explored precisely in terms of their impact on induction or protection against T2DM [30,35,38]. Accordingly, nutritional studies with the Nile rat are best accomplished by formulating specific semi-purified diets that include added supplements, or not, as the design dictates.

#### 3.2.2. Dietary Factors and T2DM

After extensive examination of the relationship between dietary factors, blood glucose, and diabetes progression, several points have been determined to date for delineating Nile rat diabetes. Many of these characteristics are illustrated by the summary of physiological data as quintiles of the RBG response from a composite group of standard control rats all fed a diabetogenic semi-purified, hiCHO diet (Diet 133 with 60:20:20, 4.2 kcal/g) for 10 weeks during several recent studies (Table 2).

First, it is important to note that diabetes induction (registered as RBG) can be demonstrated in a relatively short period (8–10 weeks) if initiated at weaning (three weeks old, body weight about 30 g) when the rats are young, growth including the pancreas is most rapid, and their glucose control mechanisms are under stress and least efficient due in part to an immature regulatory system occasioned by the shift from mother’s milk to solid lab diet. Individuals with the greatest growth rate (weight gain/day) tend to develop the most diabetes. This is especially true if the new diet presents a high glycemic load (175 per 2000 kcal diet) [30]. Under these conditions, insulin resistance can occur in a few days, but, depending on putative genetic or epigenetic factors, about half the rats prove to be ‘susceptible’ to rapid diabetes induction (i.e., a 50–60% diabetes *incidence* is observed), while the other half remain ‘resistant’ to diabetes during the 8–10-week test period when a standard hiCHO diabetogenic diet is fed (i.e., 70:10:20 or 60:20:20) following weaning at three weeks of age (Table 2). This classification of susceptibility is based on ‘*genetic permissiveness’* and has been ascertained by comparing RBG, FBG, and 30-min OGTT against several measures of diabetes recorded at necropsy for more than 1700 Nile rat necropsies. Detailed analysis revealed that RBG 75 mg/dL was the single best predictor of T2DM recorded in terminal pathologies observed after the typical 8–10-week feeding trial. All rats considered ‘non-diabetic’ or ‘resistant’ to T2DM at necropsy had RBG 75 mg/dL [30]. On the other hand, the *severity* of the diabetes is best assessed as the magnitude of the RBG elevation, ranging from 75 to 600 mg/dL.

Other useful clinical indices of diabetes induction in Nile rats are food and water intakes. The daily consumption of calories (kcal/day) shows a strong positive correlation (*r* = 0.6) with RBG in the first two quintiles, i.e., as diabetes develops, but before it becomes clinically relevant. Thus, it could be argued that growth and caloric intake both lead the diabetes (RBG) higher until ketosis becomes apparent (in 5th quintile) and food intake becomes extraordinary. Similarly, water intake can be seen to increase when RBG rises above 125 mg/dL when moderate diabetes has developed in response to a hiCHO diet (Table 2).

#### 3.2.3. Physical Parameters

Several other points are noteworthy among the metagenetic data in Table 2. First, body weight tends to increase more rapidly in those rats developing diabetes (and eating more calories), and then declines later as ketosis advances and fat reserves and muscle are catabolized. Note that the *susceptible* rats reveal selective expansion of the perirenal (Peri) and of the Brown Adipose Tissue (BAT) fat pads, but do not qualify as obese because their BMI seldom rises more that 5% above that of *resistant* rats (data not shown) and [38]. Second, FBG was found to be indicative of diabetes observed at necropsy only when it was elevated above the normal 40–60 mg/dL baseline. However, this increase in FBG seldom occurs in a typical 10-week feeding trial from weaning, only appearing when the RBG has risen into the highest range 400 mg/dL. Thus, FBG ordinarily occurs too late to be an effective early indicator, i.e., by the time it rises above 60 mg/dL, the diabetes and tissue damage have already advanced appreciably, as signaled earlier by the RBG (Table 2). On the other hand, using the elevation of 30’-OGTT into the 150–175 mg/dL range (after gavage of 225 µL/100 g rat with a solution containing 10.6 g dextrose dissolved in 6 mL water) is too sensitive and overpredicts diabetes found at necropsy in a 10-week experiment. It can be an early indicator that metabolic dysfunction and diabetes have started, but a signal of 150–175 mg/dL is not strongly associated with the disease observed in a 10-week study unless the RBG has also been elevated for a matter of weeks (Table 2) [30]. In the advanced stage of the disease, the ensuing ketosis leads to breakdown of body fat and muscle, including muscle mass (as carcass weight), which eventually causes death if left unattended. Thus, in this model it is important to monitor worsening glucose metabolism via RBG as the best index of deteriorating whole-body physiology. In terms of gross pathology, the tails of diabetic Nile rats often become scabbed and cracked with ischemic necrosis of the distal end, and the back occasionally becomes scabbed in focal areas along with eruptive dermatitis. Also, it is not uncommon for parents to chew the tails off suckling pups when disturbed.

#### 3.2.4. Diabetic Retinopathy

The Nile rat is an exceptional nutritional model for the study of diabetic retinopathy and cataracts, both of which are common in older Nile rats after a prolonged feeding of a hiCHO diet [34,100,101]. This diabetic retinopathy includes protracted accumulation of leukocytes in retinal arteries when plasma insulin levels are high and pericyte apoptosis, linked to hyperglycemia induced accumulation of Reactive Oxygen Species (ROS), is evident. Han et al. (2017) found that after a 6-month study, no difference was observed in the nicotinamide adenine dinucleotide hydride (NADH) pathway in retinal mitochondrial respiration, but sustained hyperglycemia eventually depressed the NADH pathway in diabetic Nile rats related to an increase in FBG (which in itself implies advanced diabetes). Furthermore, increased hyperglycemia was associated with a compromised outer membrane integrity of the mitochondria [101].

#### 3.2.5. Necropsy Findings

**Figure 3:**
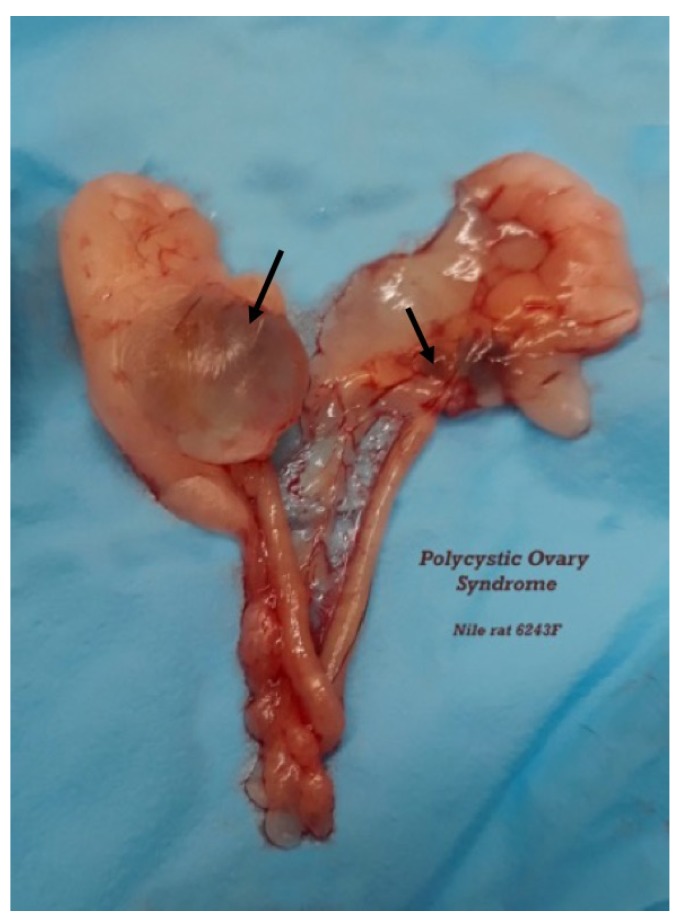
Polycystic ovaries in nulliparous 18-month-old female Nile rat fed rat chow. (RBG = 588 mg/dL and severe hyperlipidemia).

Organ weights also follow the diabetes progression (Table 2), with liver (fatty), kidney (polyuria, nephritis), and cecum enlargement (altered gut flora) corresponding to changes in glucose metabolism and hyperglycemia. Nulliparous hyperglycemic female Nile rats maintained on commercial rat chow for more than a year can develop polycystic ovaries, similar to Polycystic Ovarian Syndrome in women (Figure 3).

**Figure 4:**
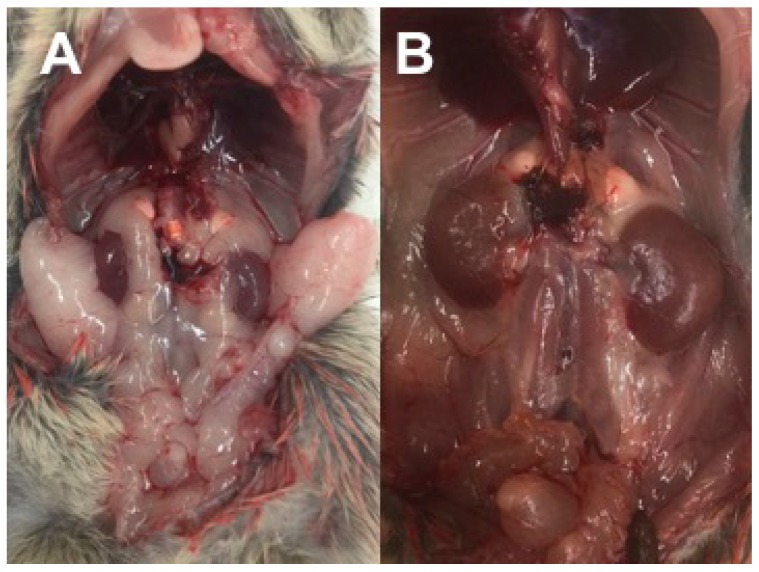
(**A**) Kidneys surrounded by Epididymal/Periovarian (Epi) and Peri fat pads; (**B**) loss of Peri fat due to ketosis exposes kidneys enlarged and swollen by chronic nephritis.

**Figure 5:**
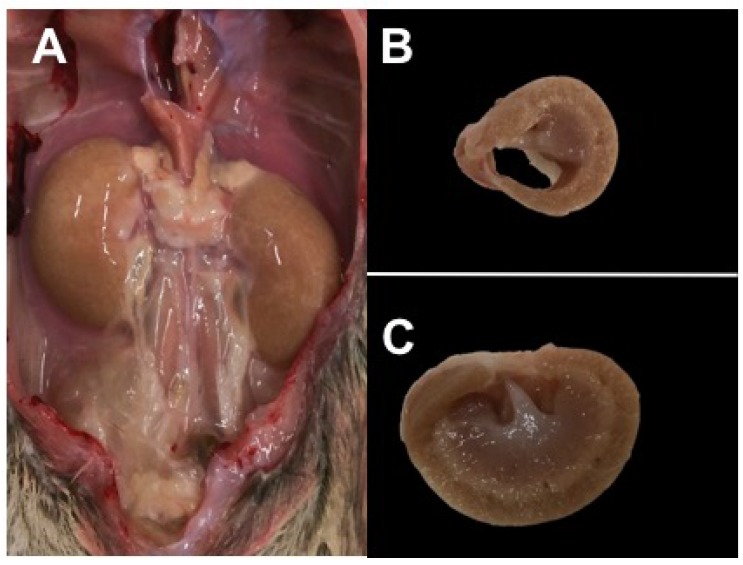
(**A**) Kidneys are enlarged and discolored due to advanced diabetes**. **Ketosis has exhausted the Peri fat pads. (**B**) Cross section of cystic dilation of diabetic kidney; (**C**) longitudinal section of diabetic kidney.

Diabetic Nile rats have a tendency to develop abdominal adiposity characterized by perirenal-retroperitoneal (Peri) fat (Figure 4A), followed by ketosis and fat-wasting in more advanced cases with loss of Peri fat (Figure 4B and Figure 5A).

The Peri fat pads and interscapular BAT fat pads seem especially sensitive, both increasing with diabetes, then collapsing rapidly as ketosis progresses. A similar pattern is seen in blood lipids, except they continue to expand with advancing T2DM as ketosis mobilizes adipose fat, and fatty acids return to the liver to exacerbate hepatic TG formation and secretion resulting in hypertriglyceridemia, and eventually hypercholesterolemia (Table 2).

Kidneys often become swollen and enlarged with surface pitting from interstitial nephritis and glomerular sclerosis. Extreme dilation of renal pelvis and nephron tubules occurs late in the disease as kidneys fail (Figure 5A–C). This is associated with increased blood urea nitrogen (BUN) and ketonuria [33,35,104].

**Figure 6:**
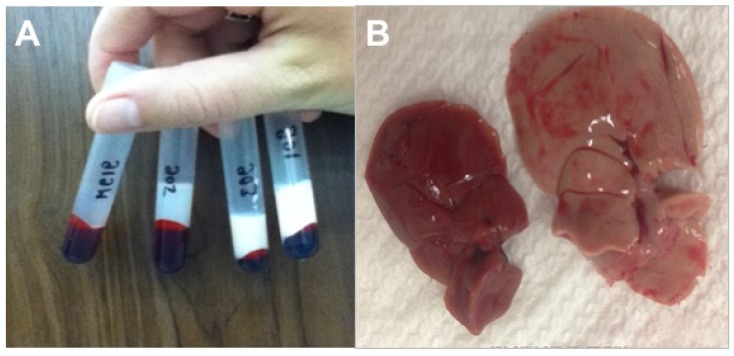
(**A**) Progressive stages of hyperlipemia in Nile rats with T2DM; (**B**) normal liver size and color (**left**) is contrasted by diet-induced hepatic enlargement and discoloration due to fatty liver (NAFLD) in advanced T2DM (**right**).

Rising RBG and marked elevation in plasma TG and cholesterol (hyperlipidemia) (Figure 6A, Table 2) is associated with non-alcoholic fatty liver disease (NAFLD). In severe ketosis, plasma TG can exceed 1000 mg/dL and cholesterol may exceed 350 mg/dL. NAFLD, also described in diabetic humans, results from hepatic TG and cholesterol accumulation (Figure 6B).

**Figure 7:**
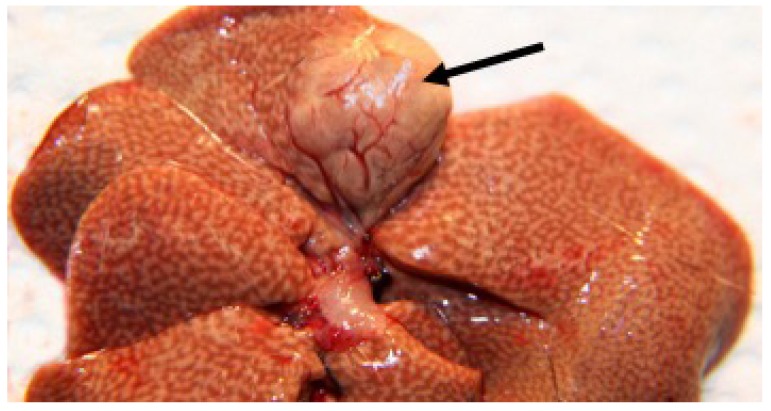
Reticulated pattern typical of fatty liver infiltration with prominent hepatic cell carcinoma (arrow) in chronic T2DM of more than a year’s duration.

In severely advanced chronic disease of several months’ duration, both cataracts and hepatic tumors (hepatoma, hepatic cell carcinoma) typically develop in more than 40% of males over a year old (Figure 7). Hepatic carcinoma in rodents has been linked to dysbiosis of the microbiome with disruption of bile acid metabolism and tumor induction by recycled secondary bile acids, specifically deoxycholic acid [105,106,107,108].

**Figure 8:**
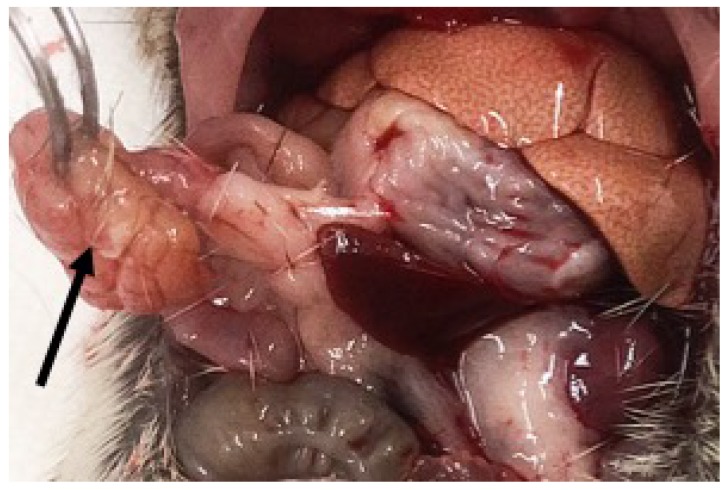
Floater—1 cm fibrous clump of necrotic adipose (in forceps, arrow) is connected to adjacent uninvolved adipose and pancreas by organized connective tissue as evidence of peripancreatic steatitis associated with ‘leaky pancreas syndrome’ occasionally observed in young rats during rapid development of T2DM when they consume a hiCHO , diabetogenic diet from weaning. It suggests a role for hiCHO diet in pancreatitis, as this is seen primarily with diets based on 70:10:20 CHO:Fat:Protein ratio with no fiber.

In addition, peripancreatic steatitis associated with ‘leaky pancreas syndrome’ is occasionally observed in young rats during rapid development of T2DM when they consume a hiCHO, diabetogenic diet from weaning. It suggests a role for hiCHO intake in pancreatitis, as this is seen in Nile rats primarily fed diets based on 70:10:20 CHO:Fat:Protein ratio with no fiber. The peripancreatic steatitis is exemplified as nodular fatty necrotic tissue, which develops into intraperitoneal ‘floaters’ when the organized, fibrous mass becomes strangulated, separates, and ‘floats’ free in the peritoneal cavity (Figure 8). With advancing diabetes, the pancreas itself becomes infiltrated and replaced by adipose tissue, such that the remaining body of the pancreas can be difficult to identify at necropsy.

#### 3.2.6. Genetic Basis of Diabetes

By way of example, genetic maps of diabetes-resistant and diabetes-prone Sand rats provided indirect evidence that a single dominant gene may interact with environmental (dietary) factors to induce the transition from normo- to hyperglycemia in that model [47]. Others have suggested that a cluster of Pdx1 genes affecting insulin secretion may have evolved in that desert rodent to help with adaptation to an arid, energy-poor environment, but no specific gene function other than Pdx1 (Pancreas and duodenal homeobox 1) transcription factor has been identified [109].

Some version of this concept might explain the genetic permissiveness apparent in the Nile rat fed a high glycemic load diet. As highlighted above, the Nile rat, like the Sand rat, can be sorted into both susceptible and resistant subgroups when consuming the same diabetogenic diet, at least during induction before discriminating gene function is overwhelmed by prolonged diet exposure and all rats become extremely diabetic (Table 2) [28,30,33,35].

#### 3.2.7. Gender and Diabetes

Unlike the Sand rat, a gender dimorphism appears in Nile rats developing T2DM, with males being more predisposed to diabetes than females. This is thought to reflect the specific effects of sex hormones because symptoms in males are exacerbated during puberty (rising testosterone), but abate somewhat in females when estrus evolves around 7–8 weeks of age. This suggests that estrogen may protect against Nile rat diabetes, which may in part be a reflection of the microbiome, as the microbiota can metabolize estrogen-like compounds into biologically active forms and promote beneficial bacteria such as Gram-positive Bifidobacterium, while suppressing harmful bacteria among the Clostridiaceae [110,111,112]. Thus, estrogen and the microbiota may act together to modulate energy metabolism, weight gain, and lipid deposition.

#### 3.2.8. Food Intake and Adipose Pools

**Figure 9:**
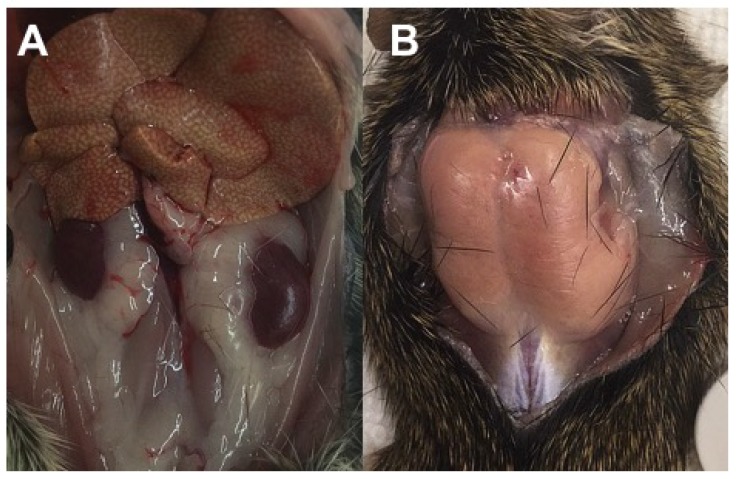
(**A**) Abundant Peri fat surrounds kidneys. Moderate NAFLD is also apparent (10-week RBG = 61). (**B**) Interscapular BAT fat pads (10-week RBG = 77).

One of the more interesting findings is that ‘susceptible’ rats fed a diabetogenic diet typically eat 10–20% more calories than ‘resistant’ rats, when the diet is carbohydrate-rich (50–70% energy as simple carbohydrate). This subtle hyperphagia may simply reflect growth demands, not necessarily loss of food intake control. A better understanding of these relationships might shed light on this aspect of human diabetes as well. Greater caloric intake is associated with expansion of the intra-abdominal fat pool, specifically the Peri fat pads surrounding the kidneys (Figure 4A, Figure 5A, and Figure 9A). This pool that correlates highly with expansion of the BAT fat pads (Figure 9B) and NAFLD, is expressed grossly as increased liver weight with the marbling and discoloration of the fatty liver (Figure 6B and Figure 7). This scenario is somewhat time-associated and self-limiting because advancing diabetes and ketosis eventually induce adipose catabolism with accompanying weight loss, despite evidence of ravenous appetite (Table 2).

BAT is thought to represent a pathway for burning excess calories when RBG becomes elevated and fat storage pools are under stress [113,114]. BAT becomes hyperplastic during T2DM induction (RBG up to 200 mg/dL), but eventually is exhausted along with all fat reserves once ketosis progresses and induces fat catabolism. The Nile rat model demonstrates that relationship well (Table 2). By increasing fat catabolism and glucose expenditure, BAT is able to better regulate blood sugar, thus making it an attractive therapeutic target for diabetes and obesity, and a good marker of fat storage under stress [30]. Chondronikola et al. (2014) found that individuals with more BAT have better fat catabolism, superior blood sugar control, and greater insulin sensitivity [115]. In fact, BAT activation appears to promote increased lipid mobilization from peripheral stores and oxidative disposal, presumably to accommodate increased fuel disposal by thermogenic BAT mitochondria [116].

**Figure 10:**
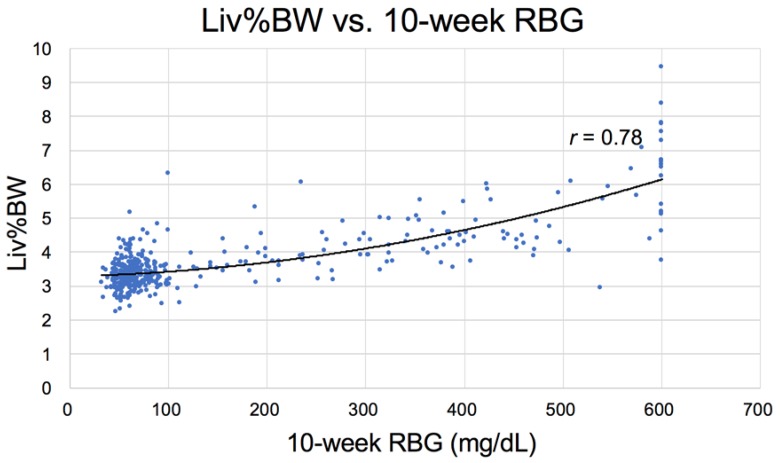
Strong correlation exists between random blood glucose (RBG) and liv%BW at necropsy (10 weeks of study) (*n* = 439). This reflects fatty liver development as diabetes progresses (see Figure 4B and Figure 5A). Blood glucose values exceeding 600 mg/dL are not recorded by the glucometer.

In Nile rats a strong positive correlation exists between RBG and liver weight expressed as percent body weight (Liv%BW), because as T2DM progresses, more glucose is converted to storage fat, in turn increasing fatty liver and liver weight (Table 2, Figure 10).

**Figure 11:**
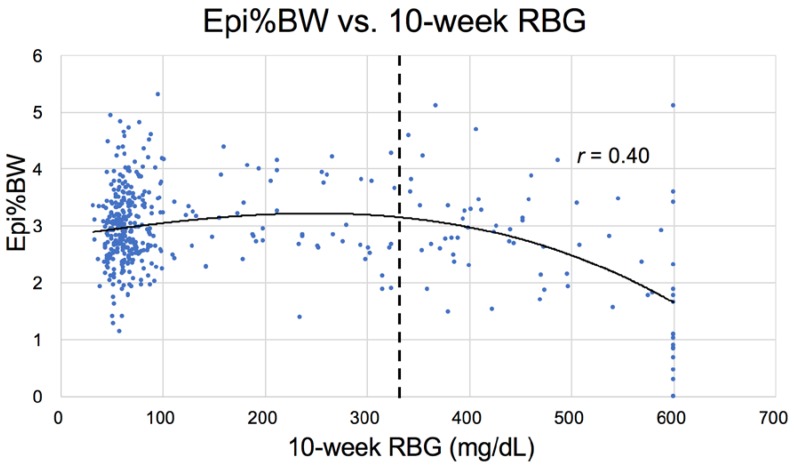
Epi fat pad %body weight (Epi%BW) plotted against random blood glucose after 10 weeks of diet from weaning (*n* = 439). Rising RBG represents the increasing likelihood of ketosis with fat loss, indicated by vertical bar and data points greater than 325 mg/dL.

**Figure 12:**
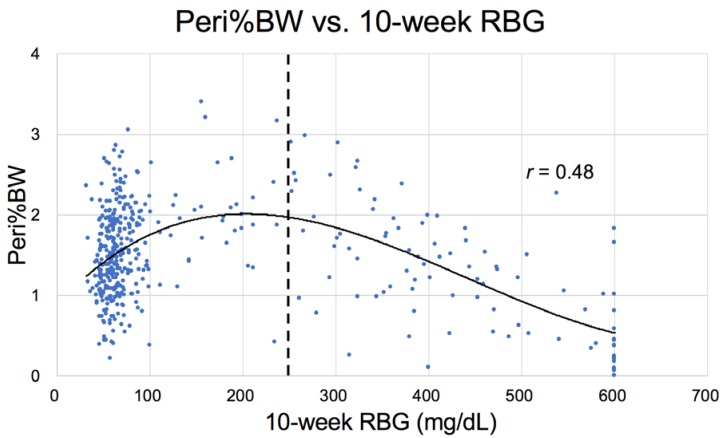
Perirenal fat pad % body weight (Peri%BW) plotted against random blood glucose (*n* = 439). The vertical bar suggests that fat loss begins sooner in this tissue mass, i.e., when RBG rises above 250 mg/dL.

In addition to the correlation between liver weight (NAFLD) and increased RBG (Figure 10) in Nile rats, a correlation also exists between an increase in RBG 75 mg/dL and the weight of the epididymal/periovarian (Epi) fat pads (Figure 11) and the Peri fat pads, but the relationship is less robust with the former (Figure 12). For example, note that the curve for perirenal fat vs. RBG (Figure 12) differs from Epi fat because the former increases initially as RBG increases, then decreases earlier and more rapidly during ketosis. The catabolic decline starts at about 250 mg/dL RBG for perirenal fat (vertical bar), compared to Epi fat (about 325 mg/dL, Figure 11), highlighting the fact that Peri fat seems more metabolically active both during induction of diabetes and during fat catabolism associated with ketosis [33]. 

**Figure 13:**
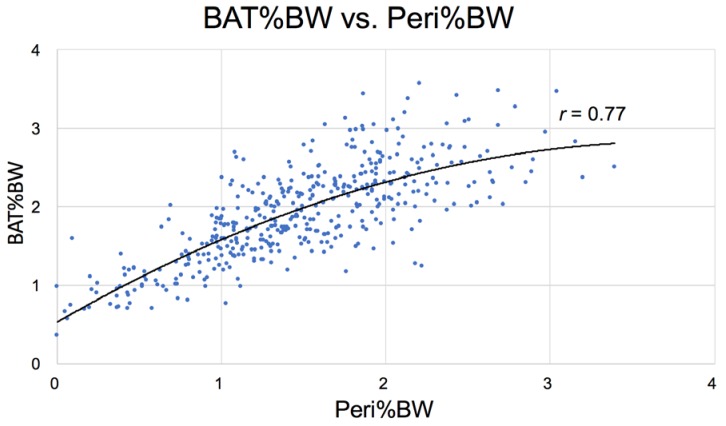
BAT%BW and Peri %BW are strongly correlated in diabetic Nile rats (*n* = 439).

**Figure 14:**
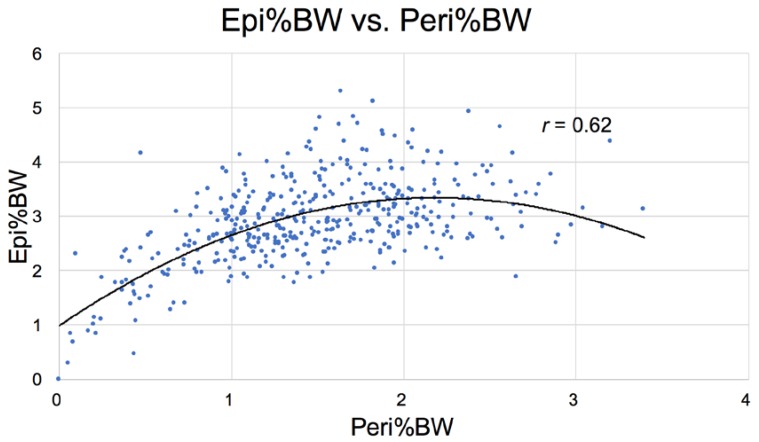
Correlation between Epi and the Peri fat pads as %BW is strong, but less robust than that between Peri%BW vs. BAT%BW (see Figure 12) (*n* = 439). This suggests that Peri and BAT are in close sync and that, though large, the Epi fat pools of Nile rat start losing their adipose storage role as diabetes progresses and may not be as involved in acute metabolic events as Peri and BAT.

The strong correlation between BAT and Peri in diabetic Nile rats suggests that these two fat pools are closely related (Figure 13) and both linked to fat dynamics of accumulation and catabolism at various stages of diabetes, more so than the correlations between other fat pools, such as the Epi fat pool (see Figure 14). BAT is the last fat store to resorb during ketosis in severe diabetes in these rats.

#### 3.2.9. Dietary Fiber

**Figure 15:**
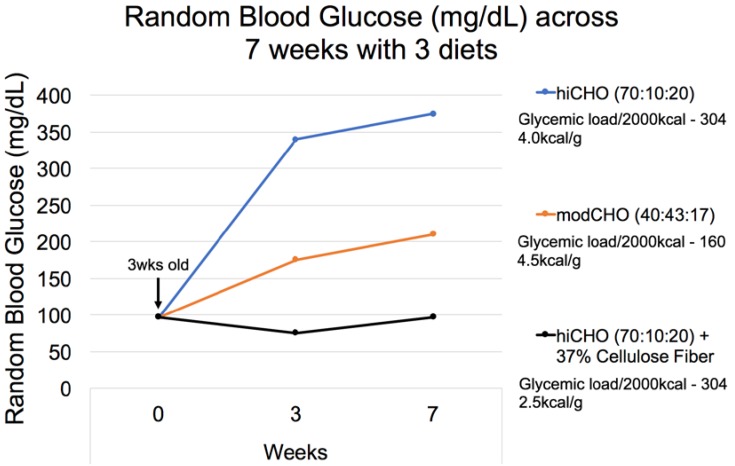
Seventy-eight male Nile rats were weaned at 3 weeks of age and split into three equal groups (Study 48): **1.** A hiCHO diet (70:10:20 CHO:Fat:Protein %energy, 4.0 kcal/g, Glycemic load 304); **2.** A modCHO diet (40:43:17, 4.5 kcal/g, Glycemic load 160); **3.** A hiCHO diet + non-fermentable fiber (70:10:20, 2.5 kcal/g, Glycemic load 304), i.e., supplemented with 37% cellulose to match the low energy saltbush diet of Sand rats. The results indicate that diet composition can definitely affect the onset and progression of T2DM, and any diet component that decreases the availability or absorption rate of glucose, including fiber, deters the diabetes in this model, emphasizing the importance of dietary fiber in the model.

As mentioned earlier, the Nile rat is carbohydrate-sensitive in terms of its diet-induced T2DM, and an increased dietary CHO:fat ratio directly impacts the severity and progression of the disease (Figure 15) [35]. Nile rats fed a high intake of refined carbohydrate (70:10:20, glycemic load at 304/2000 kcal) revealed a greater, more rapid predisposition to T2DM (both %incidence and severity) than rats fed a moderate-carbohydrate (modCHO), higher-fat diet (e.g., 40:43:17, glycemic load at 160/2000 kcal). Adding fiber further mitigated the adverse effects of the hiCHO diet as evidenced by the normoglycemic phenotype of Nile rats fed hiCHO with extremely high fiber (37%), even though a non-fermentable, insoluble form (cellulose) was supplemented. This relatively inert fiber served to dilute the simple dextrose calories, thereby lowering the rate of glucose absorption by reducing caloric density to an extremely low concentration (2.5 kcal/g diet), even though the ‘available’ glycemic load of 304/2000 kcals of diet did not change. This was designed to mimic the saltbush-Low Energy diet fed to Sand rats to prevent their T2DM [48]. The approach succeeded in preventing the diabetes, but still begs the question whether it was fiber per se, possibly fermenting somewhat in Nile rats to alter energy yield, or the displacement of simple carbohydrate to reduce the rate of glucose absorption that provided the observed benefit.

A separate study replaced the refined carbohydrates (glycemic load of 305) with natural carbohydrates as uncooked lentil flour containing crude ‘slow carbohydrates’ along with its natural soluble fermentable fiber and polyphenols (glycemic load of 102), which protected completely against the diabetes for 7 weeks. The lentil diet also maintained the 70:10:20 ratio supplied with the refined carbohydrate control, but the glycemic load was lower, causing the lentils to reduce the RBG. This was associated with an increased cecum size to suggest that enhanced carbohydrate fermentation in the large bowel helped protect against T2DM [30,35]. The microbiota in Nile rats is altered by diabetogenic diets, but especially by the presence of fiber. However, a clear relationship between specific microbiota and T2DM has not been established in this model (unpublished data). The point is that neither dietary energy density nor glycemic load per se are infallible predictors of the diabetes outcome when complex diet x gene circumstances prevail.

#### 3.2.10. Supplementation with Antidiabetic Agents and Polyphenols

Glucose metabolism can be controlled in Nile rats by common drugs such as Acarbose^TM^, which inhibits pancreatic alpha-amylase and intestinal alpha-glucosidase to block carbohydrate digestion, thus greatly reducing intestinal glucose release and absorption after a carbohydrate load. Metformin^TM^, which reduces hepatic gluconeogenesis and secretion, deters T2DM in Nile rats. This drug also alters the gut microbiome, and likely reduces peripheral insulin resistance, as well (see Table 3) [117]. Buse et al. (2016) provided evidence that a primary glucose lowering effect of Metformin^TM^ resides in the gut through its action on gut enzymes, the microbiota, and related hormones [118]. This ability of Nile rats to respond to these anti-diabetic drugs designed to control human T2DM, further emphasizes the modelling potential of Nile rats. Starlix^TM^, on the other hand, designed to stimulate insulin secretion, had no beneficial effect on diabetic Nile rats presumably because the hyperinsulinemia already present does not benefit from further insulin production.

The Nile rat has also revealed the anti-diabetic action of various polyphenols. A number of studies have shown palm fruit juice (PFJ) to be a potent anti-diabetic supplement in this model. PFJ is the water-soluble extract discarded after milling oil from the oil palm fruit. Rich in polyphenols, and shikimic acid, it has demonstrated numerous health benefits that include anti-diabetic, anti-cancer, weight loss and even anti-retroviral properties in in vitro systems and animal models [37,119]. Nile rats fed a hiCHO diet (70:10:20) supplemented with PFJ in either liquid or solid spray-dried form exhibited significant reduction in RBG, FBG and food intake (20% less energy) than control Nile rats. There was no difference in food efficiency between feeding PFJ as a drink or in food [120]. Although PFJ rich in polyphenols has proved to reduce hyperglycemia, body weight and T2DM incidence, it tends not to limit the hyperglycemia in the most genetically permissive (susceptible) rats that develop the disease on a hiCHO diet (Table 3) [120].

## 4. Conclusions and Future Perspectives

The Nile rat is an exceptional model for study of T2DM due to the parallels in the disease in terms of its etiology and progression in humans. Furthermore, the nutritional studies conducted with the Nile rat have been extensive with a clear focus on specific attributes of the diets affecting the pathophysiology of T2DM. Experience from 10 years of breeding is provided to assist the beginning researcher with details of nutrition and husbandry, including handling methods that minimize stress to the rats during diabetes induction. In summary, the Nile rat exemplifies a novel carbohydrate-sensitive model that should eventually provide invaluable insight into the role of gene x diet interactions in the development of MetS and T2DM in humans. The identification of both resistant and susceptible Nile rats consuming the same diabetogenic diet is an important development because this genetic diversity provides built-in control rats during each diet comparison, offering unique insight into the mechanisms of the disease as affected by diet.

Specifically, T2DM in Nile rats is accelerated when fed a hiCHO semi-purified, high glycemic load diet, whereas supplementation of fiber and/or polyphenols (such as PFJ) or replacing carbohydrate with either fat or protein, lessens the disease incidence and severity. Often, by the time T2DM is diagnosed in humans based on FBG, it can be late in the disease because the diabetes has already progressed to the stage of irreparable beta cell deficiency, a scenario that can be demonstrated in Nile rats, as well. This underscores the need for more specific and sensitive biomarkers to predict early onset of the disease induced by specific diet components that can then be corrected or targeted for early pharma intervention. The Nile rat offers a prime model to identify such biomarkers at the earliest time point to facilitate evaluation during future studies.

## Acknowledgments and Funding Support

A number of students and staff were involved in data collection and daily management of experiments over the years, with particular thanks to Erin Tang, Emily Lai, and Jeffery Hu, who assisted with the breeding colony maintenance and record keeping statistics. Andrew Pronczuk was involved with planning of early experiments, extensive record keeping, and necropsies. Julia Bolsinger, Andrew Auerbach, and Karen Lai also assisted with data collection related to design, daily management, and reports associated with early studies. Studies were supported in part by the Foster Lab Funds for Research and Teaching, the Malaysian Palm Oil Board, and Smart Balance, Inc. Avinaash Subramaniam was a scholar in training under a Brandeis–Massachusetts Institute of Technology (Department of Biomaterial Science and Engineering) research collaboration.

## Figures and Tables

**Table 1:** Risk indicators of Metabolic Syndrome (MetS) in humans and selected rodent models.

Model	Development of MetS		Diabetes Induced by	Signs of Metabolic Syndrome						
				Hyperphagia with Cause		Incr BP *	Abdominal Obesity	Incr TG *	Decr HDL *	Hyper Insulinemia
Human [2,3,4,5,20,21,29]	Natural (diet x gene interaction)		Carbohydrate	No	Diabetics, hiGLoad * diets	✓	✓	✓	✓	✓
Nile rat [28,30,31,32,33,34,35,36,37,38]	Natural (diet x gene interaction)		Carbohydrate	No	Diabetics, hiGLoad diets	✓	no	✓	✓	✓
Sand rat [23,24,39,40,41,42,43,44,45,46,47,48]	Selective Breeding	(diet x gene interaction)	Carbohydrate	No	Diabetics fed chow	No	✓	✓	✓	✓
C57BL/6J mouse [22,49,50,51,52,53,54,55,56,57,58,59,60,61,62,63,64,65,66]	Spontaneous Mutation	*Lep(ob);*lep deficient	Fat	No	Increased feed efficiency	✓	✓	No	No	✓
Goto-Kakizaki rat [22,67,68,69,70,71,72]	Spontaneous Mutation	*NPY mRNA* excess	Carbohydrate	✓	Leptin Resistance	No	No	No	N/A	✓
SDT rat * [73,74,75,76,77,78,79,80]	Spontaneous Mutation	*Lepr(fa)*	Carbohydrate	✓	Mutated Leptin Receptor	N/A	No	✓	N/A	✓
Koletsky rat [31]	Spontaneous Mutation	*fa(k)*	Carbohydrate	✓	Mutated Leptin Receptor	✓	✓	✓	N/A	✓
ZDF rat * [22,26,81]	Spontaneous Mutation	*fa/fa*	Fat	✓	Mutated Leptin Receptor	✓	✓	✓	✓	✓
UCD-T2DM rat/ZDSD rat * [25,26,27,82,83,84]	Selective gene mutations	fa/-, OSD *	Fat (timed +/−)	✓	Select gene mutants (fa/-)	N/A	✓	✓	Decr TC */HDL	✓

N/A—Data not available; * SDT—Spontaneously Diabetic Torii; ZDF—Zucker Diabetic Fatty; UCD-T2DM—University California Davis-Type 2 Diabetes Mellitus; ZDSD—Zucker Diabetic Sprague Dawley; OSD—Obese Sprague Dawley; BP—Blood Pressure; TG—Triglycerides; TC—Total Cholesterol; HDL—High Density Lipoprotein; hiGLoad—high glycemic load.

**Table 2:** Quintiles of RBG along with diabetes metagenetic profile for 47 male Nile Rats (3 weeks old) fed semipurified high CHO Diet 133 (60:20:20)* for 10 weeks, then subdivided into ‘resistant’ or ‘susceptible’ quintiles based on RBG 75 mg/dL, respectively. [NR Studies 130, 132, 144, 148].

Diet (CHO:Fat:Protein %energy)	Diet 133 (60:20:20) *				
	**1**	**2**	**3**	**4**	**5**
**T2DM ‘genetic permissiveness’ ranked by quintiles**	**Resist (10)**	**Resist (10)**	**Suscept (9)**	**Suscept (9)**	**Suscept (9)**
**RBG (range) after 10 weeks (mg/dL)**	**(48-61)**	**(62-69)**	**(71-143)**	**(183-424)**	**(427-600)**
**ave Random Blood Glucose (mg/dL) after 10weeks**	54 ± 5 ^ab^	66 ± 3 ^cd^	96 ± 27 ^ef^	269 ± 77 ^aceg^	535 ± 73 ^bdfg^
**Body Weight (g) after 10 weeks**	97 ± 9 ^ab^	102 ± 7 ^c^	105 ± 4 ^ad^	108 ± 4 ^be^	92 ± 10 ^cde^
					
**Food Intake in 9th week (kcal/d)**	31.8 ± 2.5 ^a^	34.2 ± 2.4 ^b^	34.7 ± 1.3 ^b^	35.2 ± 1.1 ^d^	43.8 ± 10.2 ^abcd^
					
**Water Intake in 9th week (mL/week)**	29 ± 7 ^a^	29 ± 7 ^b^	35 ± 10 ^c^	43 ± 22 ^d^	257 ± 289 ^abcd^
					
**Oral Glucose Tolerance Test (mg/dL) after 10 weeks**					
Fasting Blood Glucose (FBG) 0 min	50 ± 11 ^a^	49 ± 8 ^b^	47 ± 16 ^c^	44 ± 6 ^d^	92 ± 57 ^abcd^
30 min	143 ± 49 ^abcd^	223 ± 58 ^ae^	257 ± 105 ^bf^	270 ± 49 ^cg^	490 ± 89 ^defg^
					
**Organ weight (%BW)**					
Liver	3.83 ± 0.67 ^a^	3.46 ± 0.17 ^b^	3.53 ± 0.22 ^c^	3.99 ± 0.76 ^d^	5.94 ± 1.17 ^abcd^
Kidney	0.70 ± 0.04 ^a^	0.68 ± 0.04 ^b^	0.68 ± 0.04 ^c^	0.78 ± 0.06 ^d^	1.22 ± 0.27 ^abcd^
Cecum	1.10 ± 0.42 ^a^	1.16 ± 0.21 ^b^	1.09 ± 0.17 ^c^	1.06 ± 0.19 ^d^	2.06 ± 0.65 ^abcd^
Adipose					
Epididymal	3.15 ± 0.70 ^a^	3.27 ± 0.39 ^b^	3.07 ± 0.50 ^c^	2.92 ± 0.69 ^d^	1.92 ± 0.86 ^abcd^
Perirenal	1.46 ± 0.33 ^abcd^	2.08 ± 0.52 ^ae^	2.03 ± 0.49 ^bf^	2.09 ± 0.48 ^cg^	0.68 ± 0.56 ^defg^
Brown fat	1.90 ± 0.39 ^abcd^	2.35 ± 0.57 ^ae^	2.68 ± 0.46 ^bf^	2.38 ± 0.43 ^cg^	1.10 ± 0.42 ^defg^
Total fat	6.51 ± 1.00 ^a^	7.01 ± 1.37 ^b^	7.42 ± 1.68 ^c^	6.66 ± 1.63 ^d^	3.70 ± 1.71 ^abcd^
					
**Plasma**					
Cholesterol (mg/dL)	126 ± 25 ^a^	124 ± 21 ^b^	125 ± 24 ^c^	145 ± 46 ^d^	482 ± 524 ^abcd^
Triglycerides (mg/dL)	63 ± 24 ^a^	71 ± 25 ^b^	73 ± 24 ^c^	110 ± 38 ^d^	292 ± 401 ^abcd^

* Represents CHO:Fat:Protein %energy with Glycemic load of 224/2000 kcal; 4.2 kcal/g dry diet; Values are mean ± SD; a, b…Means in a row sharing common superscripts are significantly different (*P* 0.05) by one-way ANOVA and Fisher’s PLSD test; ^#^ *n* = 9–10 per Quintile, total 47 rats.

**Table 3:** Diabetic profile of 53 male Nile Rats (3 weeks old) fed semipurified high CHO diet (70:10:20) * for 7 weeks, with diabetes ‘resistant’ or ‘susceptible’ rats based on RBG 75 mg/dL.

**CHO:Fat:Protein %Energy**	**70:10:20**		**70:10:20**		**70:10:20**		**70:10:20**		**70:10:20**	
**Supplement**	None		0.075% Starlix^TM^ (0.19 mg/kcal)		0.05% Acarbose^TM^ (0.13 mg/kcal)		0.25% Metformin^TM^ (0.63 mg/kcal)		10% PFJ (1.4 mgGAE/kcal) **	
**7 week RBG 75 mg/dL T2DM**	**75**	**75**	**75**	**75**	**75**	**75**	**75**	**75**	**75**	**75**
	**resist**	**suscept**	**resist**	**suscept**	**resist**	**suscept**	**resist**	**suscept**	**resist**	**suscept**
**(n, %Incidence T2DM) **	5	7 (58%)	4	8 (67%)	4	1 (20%)	5	1 (17%)	10	2 (17%)
										
**Random Body Weight (g)**										
Initial (3 weeks of age)	31 ± 6	37 ± 4 ^abcd^	28 ± 7 ^a^	34 ± 4	36 ± 9	25 ± 0	31 ± 6 ^b^	23 ± 0	31 ± 5 ^c^	27 ± 1 ^d^
After 4 weeks	74 ± 9	72 ± 9	71 ± 14	78 ± 9	65 ± 5	77 ± 0	66 ± 4	79 ± 0	69 ± 8	69 ± 8
After 7 weeks	79 ± 19	83 ± 8	80 ± 14	86 ± 8	75 ± 6 ^a^	80 ± 0	84 ± 7	98 ± 0	88 ± 7 ^a^	83 ± 18
**Body Weight gain/day**	0.98 ± 0.37	0.94 ± 0.22	1.06 ± 0.27	1.06 ± 0.17	0.80 ± 0.26	1.12 ± 0.00	0.94 ± 0.06	1.19 ± 0.00	1.02 ± 0.13	1.00 ± 0.33
After 4 weeks	70 ± 17 ^ab^	178 ± 138 ^acdef^	73 ± 21 ^c^	199 ± 120 ^bhij^	79 ± 12 ^dh^	111 ± 0	70 ± 19 ^ei^	57 ± 0	74 ± 11 ^fj^	90 ± 14
After 7 weeks	61 ± 10 ^ab^	239 ± 171 ^acdefg^	58 ± 6 ^ch^	247 ± 197 ^bh^	63 ± 6 ^d^	104 ± 0	64 ± 8 ^e^	204 ± 0	55 ± 8 ^fg^	152 ± 85

* Diet represents CHO:Fat:Protein %energy with Glycemic Load of 304/2000 kcal, 4.0 kcal/g. Values are mean ± SD; ** PFJ supplemented at 5.4 mgGAE/g diet; a, b Means in a row sharing common superscripts differ (*P* 0.05) by one-way ANOVA and Fisher’s PLSD test; Starlix^TM^ (Nateglinide, Novartis); Acarbose^TM^ (Bayer Pharmaceuticals); Metformin^TM^ (Glucophage, Bristol-Myers Squibb).
